# Statistical analysis plan for the PlAtelet Transfusion in Cerebral Haemorrhage (PATCH) trial: a multicentre randomised controlled trial

**DOI:** 10.1186/s13063-016-1478-y

**Published:** 2016-08-02

**Authors:** M. Irem Baharoglu, Rustam Al-Shahi Salman, Charlotte Cordonnier, Rob J. de Haan, Yvo B. W. E. M. Roos

**Affiliations:** 1 Department of Neurology, Academic Medical Centre, H2-222, PO Box 22660, 1100 DD Amsterdam, The Netherlands; 2 Centre for Clinical Brain Sciences, University of Edinburgh, Chancellor’s Building, Royal Infirmary of Edinburgh, 49 Little France Crescent, Edinburgh, EH16 4SB UK; 3 Univ. Lille, Inserm U1171, Degenerative & Vascular Cognitive Disorders, CHU Lille, Department of neurology, F-59000 Lille, France; 4 Clinical Research Unit, Academic Medical Centre, PO Box 22660, 1100 DD Amsterdam, The Netherlands

**Keywords:** Stroke, Intracerebral haemorrhage, Platelet transfusion, Haemostatic drugs, Randomised controlled trial, Statistical analysis plan

## Abstract

**Background:**

Use of antiplatelet therapy shortly before stroke due to spontaneous primary intracerebral haemorrhage (ICH) is associated with higher case fatality in comparison to ICH without prior antithrombotic drug use. The PlAtelet Transfusion in Cerebral Haemorrhage (PATCH) trial aimed to assess the effect of platelet transfusion in patients presenting with ICH while using antiplatelet therapy. The main hypothesis of PATCH was that platelet transfusion would reduce death or dependence by reducing ICH growth.

**Methods/Design:**

PATCH was a multicentre prospective, randomised, open, blinded endpoint (PROBE) parallel group trial, conducted at 60 hospitals in The Netherlands, Scotland and France. Forty-one sites enrolled 190 patients with spontaneous supratentorial ICH aged ≥18 years, who had used antiplatelet therapy for ≥7 days preceding ICH, if Glasgow Coma Scale was ≥8. Participants were randomised (1:1, with a secure web-based system using permuted blocks, stratified by study centre and type of antiplatelet therapy pre-ICH) to receive either platelet transfusion within 6 hours of symptom onset and 90 minutes of diagnostic brain imaging, or standard care without platelet transfusion. The primary outcome was modified Rankin Scale (mRS) score assessed blind to treatment allocation at 3 months after ICH. Planned secondary outcomes included ICH growth on brain imaging performed approximately 24 hours after randomisation, survival at 3 months, disability at 3 months scored using the Amsterdam Medical Centre linear disability score, heterogeneity of treatment effect on mRS and ICH growth according to presence of the computed tomography angiography spot sign, causes of poor outcome, and cost-effectiveness. Safety outcomes were transfusion reactions, thromboembolic complications, and serious adverse events occurring during hospitalisation. This statistical analysis plan was written without knowledge of the unblinded data.

**Trial registration:**

The trial was registered with the Netherlands Trial Register on 29 April 2008 (NTR1303).

## Update

### Background

Haemorrhagic stroke causes 11 % of all new strokes in high-income countries and 22 % in low-middle income countries [1], yet the 2013 Global Burden of Disease study estimated that haemorrhagic stroke accounted for half of all stroke deaths and approximately 47 million (42 %) of the disability-adjusted life-years lost due to stroke [2]. Spontaneous (non-traumatic) intracerebral haemorrhage (ICH) caused by cerebral small vessel diseases (so-called ‘primary’ ICH) accounts for two-thirds of haemorrhagic stroke [3]. ICH causes >2 million strokes per year, and more than one quarter of patients with incident ICH in high-income countries are already taking antiplatelet drug therapy at the time of ICH [4–6].

In a systematic review and meta-analysis of 25 cohort studies, antiplatelet therapy use at the time of ICH was associated with an increase in the risk of death (multivariable adjusted odds ratio [OR] 1.27, 95 % confidence interval [CI] 1.10 to 1.47) in comparison to patients with ICH not taking antithrombotic drugs [7]. In observational analyses, pre-ICH antiplatelet therapy use and reduced platelet activity have been associated with early ICH volume growth [8, 9], which is one of the most important determinants of poor outcome after ICH [10].

Consequently, for people taking antiplatelet therapy shortly before ICH, restoration of platelet function may improve haemostasis, reduce ICH growth, and thereby improve outcome. Observational studies have varied in their support for this hypothesis [11–16], but randomised controlled trials have not tested the effects of platelet transfusion on clinical outcomes for people with acute ICH associated with the use of antiplatelet therapy [16, 17].

Therefore, we designed the PlAtelet Transfusion in Cerebral Haemorrhage (PATCH) randomised controlled trial to test the hypothesis that for people with acute ICH associated with the use of antiplatelet therapy, platelet transfusion compared to standard care without platelet transfusion reduces death or dependence. One other similar trial is ongoing (NCT00699621). The PATCH trial has been registered (Netherlands Trial Register NTR1303), its protocol has been published [18], and we now describe the final statistical analysis plan, which was written and submitted without knowledge of the outcome data.

### Objectives

#### Primary objective

The primary aim of the PATCH trial was to investigate whether platelet transfusion reduced poor outcome at 3 months after ICH affecting patients who were using antiplatelet therapy at the time of ICH. The poor outcome of death or dependence was defined in the protocol as a modified Rankin Scale (mRS) score of 4 to 6 [18].

#### Secondary objectives

The secondary aims of the PATCH trial were to investigate whether platelet transfusion: was safe; decreased ICH growth; improved survival at 3 months; reduced poor outcome across the full ordinal scoring range of the mRS at 3 months; reduced poor outcome defined as mRS scores 3 to 6 at 3 months; and reduced disability at 3 months scored using the Amsterdam Medical Centre linear disability score (ALDS). We aimed to perform a sub-study to determine whether the presence of the ‘spot sign’ on computed tomography angiography (CTA) at baseline modified the effect of platelet transfusion on the primary outcome and ICH growth [19]. We also intended to describe the causes of poor outcome and cost-effectiveness of the intervention [18].

### Design

PATCH was a multicentre prospective, randomised, open, blinded endpoint (PROBE) parallel group trial conducted at 60 hospitals in The Netherlands, Scotland, and France. Between 4 February 2009 and 8 October 2015, 41 sites enrolled 190 patients with spontaneous supratentorial ICH aged ≥18 years, who had used antiplatelet therapy for ≥7 days preceding ICH, if Glasgow Coma Scale was ≥8. Participants were randomised in a 1:1 ratio to receive either platelet transfusion within 6 hours of symptom onset and 90 minutes of diagnostic brain imaging, or standard care without platelet transfusion. Allocation concealment was ensured by a secure web-based system using permuted blocks, stratified by study centre and type of antiplatelet therapy pre-ICH (cyclooxygenase inhibitor only, adenosine diphosphate (ADP) receptor inhibitor only, cyclooxygenase inhibitor in combination with an adenosine reuptake inhibitor, or cyclooxygenase inhibitor in combination with ADP receptor inhibitor). Patients and investigators were not blinded to the allocated treatment group, but the primary outcome was assessed blind to treatment allocation at 3 months after ICH.

#### Inclusion criteria

1. Age ≥ 18 years
2. Non-traumatic, supratentorial ICH confirmed by brain imaging
3. Glasgow Coma Scale score 8–15
4. Antiplatelet therapy with a cyclooxygenase inhibitor (aspirin or carbasalate calcium), or ADP receptor inhibitor (clopidogrel), or an adenosine reuptake inhibitor (dipyridamole) used for at least the 7 days preceding ICH
5. Treatment can be initiated within 6 hours of symptom onset and within 90 minutes of brain imaging
6. Pre-stroke modified Rankin Scale score of 0 (no symptoms) or 1 (no significant disability despite symptoms; able to carry out all usual duties and activities)

#### Exclusion criteria

1. Haematoma on brain imaging suggestive of epidural or subdural haematoma, or an underlying aneurysm or arteriovenous malformation
2. Planned surgical evacuation of haematoma within 24 hours after admission
3. Presence of intraventricular blood more than sedimentation in the posterior horns of the lateral ventricles
4. Previous adverse reaction to platelet transfusion
5. Known use of vitamin K antagonists [unless international normalised ratio (INR) ≤1.3]
6. Known thrombocytopenia < 100 x 10^9^/L
7. History of coagulopathy
8. Mental incapacity, by legal criteria, before stroke
9. Death appears imminent
10. No written informed consent

#### Intervention

Patients were assigned in a 1:1 ratio to receive either standard care + platelet transfusion or standard care alone. Platelet transfusion had to be initiated within 6 hours after onset of ICH symptoms and within 90 minutes of brain imaging. Platelets were supplied by national or regional blood supply organisations, issued by the hospital transfusion laboratory, and transfusion was carried out according to local transfusion protocols. Patients using aspirin/carbasalate calcium or adenosine reuptake inhibitor without an ADP receptor inhibitor were given either a single buffycoat-derived leucocyte-depleted platelet adult dose equivalent unit or a single leucocyte-depleted platelet unit. Patients using ADP receptor inhibitors, either alone or in combination with other antiplatelet therapy, were given two units.

Standard care was not defined, but was given according to European [20] and national guidelines of the time [21, 22].

#### Data collection

Investigators were asked to record patient characteristics and demographics before randomisation, which only required the patient’s age and pre-ICH antiplatelet therapy to be specified. Brain computed tomography (CT) or magnetic resonance imaging (MRI) ± angiography was performed in routine clinical practice at hospital admission. Repeat brain imaging was performed as a trial procedure and was intended to be the same modality as baseline scan, done at 24 ± 3 hours after randomisation. Diagnostic and 24-hour brain imaging studies were obtained in DICOM format from trial sites, and analysed centrally in Amsterdam. Investigators collected information about the occurrence of serious adverse events/reactions during hospital admission and the date and destination of discharge. Neurologists or research nurses who were trained and blinded to treatment allocation collected follow-up data at 3 months after randomisation by use of either structured telephone interview or face-to-face interview. Data were collected centrally in the different participating countries. Good clinical practice (GCP)-compliant internet-based remote data capture (Oracle Clinical, Redwood Shores, CA, USA) was used for entering, managing, and validating the data from the sites.

#### Primary outcome

- Poor outcome (death or dependence), defined as a mRS score of 4 to 6, at 3 months after randomisation.

#### Secondary outcomes

- Safety of platelet transfusion (see Safety below)
- ICH growth, defined as the absolute difference in intraparenchymal ICH volume (mL) between brain imaging at baseline and repeat imaging after 24 ± 3 hours, assessed by automated planimetric software, checked by two independent radiologists for accuracy
- Survival at 3 months
- The full ordinal score range of mRS at 3 months
- Poor outcome defined as mRS 3–6 at 3 months
- Disability at 3 months scored using the Amsterdam Medical Centre linear disability score (ALDS)
- Presence of the ‘spot sign’ on CTA at baseline and whether this modified the effect of platelet transfusion on the primary outcome and ICH growth
- Causes of poor outcome
- Cost-effectiveness of platelet transfusion

#### Safety

Investigators recorded all adverse events during hospitalisation and reported any adverse event that was considered to be a serious adverse event (SAE) if it resulted in death, was life threatening (at the time of the event), required prolongation of hospitalisation, or resulted in persistent or significant disability or incapacity, or any other important medical event. Safety outcomes were classified as:

- Complications of ICH (ICH growth, brain oedema, brain herniation, intraventricular extension, hydrocephalus)
- Thromboembolic complications (cerebral infarction, myocardial infarction, systemic embolism, pulmonary embolism)
- Transfusion reactions (non-haemolytic, anaphylactic, transfusion-related acute lung injury, post-transfusion purpura, graft-versus-host disease, transfusion-transmitted bacterial infection)
- Other (infection – urinary tract or pulmonary – and epileptic seizures)

### Statistical methods specified in the protocol

#### Sample size calculation

We estimated that at least 70 % of participants would have poor outcome (mRS 4 to 6) [18]. We aimed for an absolute reduction in the risk of this poor outcome by 20 % to 50 %. A two-group chi-squared test with a 0.05 two-sided significance level would have 80 % power to detect a 20 % absolute risk difference between a standard care group of 95 patients in which 70 % experienced poor outcome and a platelet transfusion group of 95 patients in which 50 % experienced poor outcome at 3 months (OR = 0.43). With this sample size, a two-sided 95 % CI for the difference between the proportions would extend 13.6 % from the observed difference in proportions.

#### Proposed analyses

Baseline characteristics will be summarized using simple descriptive statistics. The main analysis of the PATCH trial was going to consist of a single comparison between the trial treatment groups of the primary outcome after 3 months (dichotomised mRS score 0–3 vs. 4–6). The analysis will be based on the intention-to-treat principle. The effect size will be expressed in a relative risk estimates and absolute risk reduction. Additionally the primary outcome will be analysed using multivariable logistic regression, adjusting (if necessary) for clinically relevant baseline imbalances and treatment characteristics. The effect size will be expressed in an adjusted OR. With regard to the range of secondary outcome parameters we will use simple 2 × 2 tables, two-group *t* tests, Mann-Whitney *U* tests, and multivariable linear and logistic regression models, when appropriate. With respect to the primary outcome predefined subgroup analyses will be performed: (a) pre-ICH antiplatelet therapy, (b) treatment within 2.5 hours versus 2.5–6 hours after symptom onset. In all analyses, statistical uncertainty will be quantified with 95 % CIs.

### Interim analyses and safety reporting

The Data Safety Monitoring Board (DSMB – see Appendix) consisted of three independent trial experts (one statistician, one neurologist, and one expert in vascular medicine) to monitor safety and perform an analysis of unblinded effectiveness data at one time point after inclusion of 100 patients in the trial. At the time of inclusion of the 100^th^ patient, SAEs needed to be recorded and reported to the trial coordinating centre, and the DSMB needed to be reconstituted (Appendix), by which time 160 patients had been included in the trial and an interim analysis could be performed on the first 154 patients for whom both outcome and SAE data were available. The DSMB were provided with a report prepared by an independent statistician that included baseline variables [gender, age, antiplatelet therapy, and National Institutes of Health Stroke Scale (NIHSS) score], SAEs, the primary outcome, and a distribution of the mRS in each of the arms of the trial unblinded to the treatment group. The DSMB was instructed in a charter to look at safety (deaths and the number of SAEs in both groups) and efficacy (primary outcome, dichotomised 0–3 vs. 4–6, using a Haybittle-Peto stopping rule with a *p* value set at 0.001 [23]). The DSMB assessed the unblinded data in a closed session, independent of the investigators, in October 2015. The DSMB had not been provided with a pre-specified threshold for futility. Additionally the DSMB was asked to advise the executive committee on possible continuation of the trial beyond its pre-specified sample size if analyses showed a possible signal of efficacy. By the time the DSMB gave its final advice to the executive committee, the trial had just reached its pre-specified sample size of 190 patients. The verdict of the DSMB was that their advice was without consequence for the study and to not include more patients beyond the pre-specified sample size. There was a separate DSMB in France that performed ongoing safety monitoring for patients included in France (see Appendix).

## Statistical analysis plan

### Overall principles

The data analysis will start after the 3-month follow-up data of the last included patient has been obtained, and after the clinical trial module of the study database has been cleaned and locked.

The analyses will be done by a co-investigator (MIB) supervised by the principal investigator (YBWEMR) and an independent epidemiologist/statistician of the Amsterdam Medical Centre Clinical Research Unit. The statistical programming and analysis to produce all summary tables and figures will use the statistical package IBM SPSS statistics version 22 (IBM Corp., Armonk. NY, USA).

In general, variables will be summarised using simple descriptive statistics such as means with standard deviation for continuous symmetric variables, medians and interquartile ranges for continuous skewed variables, and frequencies with percentages for categorical variables.

All analyses will be done according to the intention-to-treat principle, by analysing patients in the groups to which they were allocated by randomisation. The analyses will first be performed blind to treatment allocation, to allow for checking of the data and the proposed summaries/analyses. After the investigation and correction of any isolated or systematic data errors, treatment allocation will be unmasked. The primary outcome will be analysed in the pre-specified subgroups below, irrespective of the presence of statistical significance in the overall analysis. Safety outcomes will be additionally analysed in the as-treated (not per protocol) population.

#### Overall level of statistical significance

According to Haybittle-Peto’s stopping rule, no adjustment of the *p* value will be used for the final analysis [23]. A two-sided *p* value < 0.05 will be considered statistically significant. Statistical uncertainty will be expressed in a two-sided 95 % CI.

#### Handling of missing data

Missing baseline and outcome data will not be imputed. We will state which data are missing and calculate frequencies using the total number of patients with available data. When a patient is lost to follow-up or has withdrawn consent, we will use all available data up until withdrawal of consent or loss to follow-up. A specific section in the paper will report on missing data.

### Definition of populations for analysis

The unit of analysis will be the patient.

#### Intention-to-treat population

All randomised patients will be analysed in the treatment group to which they were originally allocated irrespective of non-adherence or deviations from protocol. Occasionally, investigators randomised the same patient twice in the web-based randomisation system because they were not aware of the treatment allocation from the first randomisation; in these cases, the treatment allocation of the second randomisation was used in practice, and will be used in the final analyses, and the first unused randomisation record was removed from the trial database.

#### As-treated population

Patients will be analysed in groups according to treatment received irrespective of allocated treatment at randomisation, thus creating a group that did not receive platelet transfusion (control) and a group that received any platelet transfusion (intervention). The patients will still be included in the as-treated analysis if there was a protocol violation (e.g. not receiving treatment within the described time frame, not receiving the correct number of units of platelets, or not meeting inclusion or exclusion criteria).

### List of analyses

#### Recruitment and retention

The trial profile and inclusion will be shown in a Consolidated Standards of Reporting Trials (CONSORT) flow diagram, including the total number of randomised patients and then showing per treatment group the numbers receiving allocated treatment, withdrawing consent, and lost to follow up.

#### Baseline characteristics

The baseline characteristics of all participants in each treatment group will be outlined in a table, but no formal statistical testing will be performed. The table will describe the following variables: age, sex, vascular co-morbidities, history of coagulopathy, type of antiplatelet therapy (as used for stratification), use of a statin, Glasgow Coma Scale score, NIHSS score, platelet count, ICH location and volume on baseline imaging, presence/extent of intraventricular blood on baseline imaging, and the ICH score [24].

#### Protocol deviations and violations

All substantial protocol violations will be listed.

#### Adherence to allocated treatment

Adherence will be reported descriptively.

#### Primary outcome

We have chosen to quantify the effect of platelet transfusion on the primary outcome of the mRS at 3 months with the common OR and 95 % CI from an ordinal logistic regression analysis of the shift of all categories of the mRS [25], rather than using the pre-specified fixed dichotomous approach. We have explained our rationale for this below. First a “crude” OR with 95 % CI will be calculated after which multivariable regression analysis will be used to adjust for the following:

- ▪ Type of antiplatelet therapy used (stratification variable: cyclooxygenase inhibitor only vs. ADP receptor inhibitor only vs. cyclooxygenase inhibitor in combination with an adenosine reuptake inhibitor vs. cyclooxygenase inhibitor in combination with ADP receptor inhibitor).
- ▪ ICH severity, quantified by the ICH score [24]
- ▪ Any large, chance imbalances at baseline between intervention and control groups in covariates that might have a major influence on the primary outcome.

We will not correct for the number of centres that recruited patients, although this was a stratification variable. This because many centres included only a small number of patients and adjusting for all centres might lead to unreliable estimates of the treatment effect. We will perform a separate sensitivity analysis to evaluate modification of treatment effect by centre, as described below.

#### Secondary outcomes

Survival of patients and the proportion of patients with poor outcome according to the different fixed dichotomous analyses of the mRS (score 4–6 or 3–6), at 3 months will be expressed as proportions for each treatment group and the difference will be tested using the chi-square test and quantified using OR with 95 % CI. ICH volume will be expressed in absolute mL and the difference in this volume (i.e. growth) between imaging at baseline and after 24 hours will be calculated in mL, where a positive figure will represent growth and a negative value will represent shrinkage of ICH. The median difference in ICH growth will be compared between treatment groups using the Mann-Whitney *U* test. The ordinal distribution of the mRS will be shown for patients that received platelet transfusion within 3 hours and between 3 and 6 hours of symptoms separately.

#### Safety outcomes

Safety outcomes will be reported in the intention-to treat and as-treated populations. Proportions will be tested for between-group differences using the chi-square test and expressed in OR with 95 % CI. For all continuous variables either means with standard deviation or medians with interquartile range will be calculated where appropriate and testing for difference will be performed with either the two-group *t* test or Mann-Whitney *U* test where appropriate.

#### Subgroup analyses

We will test for modification of the effect of platelet transfusion on the primary outcome using ordinal regression analyses for the following subgroups:

- ▪ Pre-ICH antiplatelet therapy used: single vs. dual therapy
- ▪ ICH volume at baseline, trichotomised according to the distribution of ICH volume on the baseline diagnostic scan
- ▪ country of randomisation (The Netherlands vs. Scotland vs. France)

#### Sensitivity analyses

Using ordinal regression we will test for heterogeneity in the effect of platelet transfusion on the primary outcome in the centres that randomised at least five patients (eight in The Netherlands, three in Scotland, and three in France), representing 66 % of all patients included. Inclusion of at least five patients was chosen so that it was likely that there would be at least one outcome in each treatment arm of the trial in each centre.

## Differences between the protocol and the statistical analysis plan

The principal difference from the analysis plan specified in the trial protocol [18] is the analysis of the primary outcome (mRS) using ordinal logistic regression to calculate the common OR (which was originally specified as a secondary analysis), rather than a fixed dichotomous analysis (originally specified as a primary analysis). We have made this decision for several reasons. First, common practice in the analysis of ordinal scales like the mRS as the primary outcome in stroke trials has evolved to use the ordinal logistic regression approach, because it is more statistically efficient/powerful, and treatments for acute stroke (like platelet transfusion) are likely to shift all patients somewhat on a functional outcome scale [25]. Second, recent trials have illustrated this point, by demonstrating a small but clinically meaningful effect of thrombolysis after acute ischaemic stroke [26] and intensive blood pressure lowering after ICH [27] on the mRS by using ordinal logistic regression, but not the fixed dichotomous approach. Third, the PATCH trial is particularly likely to benefit from this approach, as a small trial with a sample size of 190. The power to detect a common OR of 0.43 (as in the original sample size calculation) in a shift analysis of all pairs of mRS categories would increase from 80 % to 91 % with a sample size of 190 patients. For this calculation we assumed that the distribution of the mRS in the control arm would be similar to that in the STICH II trial [28], which had a similar proportion of patients with poor outcome as assumed in our original sample size calculation.

Furthermore, there are several differences in the planned secondary outcomes: ICH volume was calculated using an automated planimetric method, rather than the previously described ABC/2 formula. We had intended to perform a larger sub-study of the spot sign on CT angiography, but this was performed infrequently in clinical practice, making this sub-study too small to generate a statistically meaningful analysis of the modification of treatment effect by the spot sign. Similarly, the platelet function sub-study was only performed in a few centres in France, precluding any analyses. Funding was insufficient for the initial planned investigation of causes of poor outcome, the assessment of functional outcome using the Amsterdam Medical Centre linear disability score, and the collection of economic-related data for the purpose of cost-effectiveness analysis. Description of the distribution of the mRS in patients that received platelet transfusion within 3 hours vs. 3 through 6 hours of symptoms was added, instead of a planned subgroup analysis according to timing of treatment. This because timing of treatment was only recorded for those patients that received platelet transfusion precluding a formal subgroup analysis.

Lastly, the stratification factor “recruiting centre” could not be included as a variable to adjust for in the primary analysis because many centres included a small number of patients, which could lead to unreliable estimates of the treatment effect. To explore the impact of this variable on the primary outcome, we opted to perform a separate sensitivity analysis at a subset of centres recruiting five or more patients.

## Current trial status

PATCH trial sites recruited from 4 February 2009 in The Netherlands, from 23 May 2011 in Scotland, and from 28 September 2013 in France, and the last patient was randomised on 8 October 2015. The last follow-up was obtained on 6 January. At the time of writing, the trial data have been cleaned and checked for completeness and internal consistency, blinded to treatment allocation. The database will only be locked after this statistical analysis plan has been date and time stamped in a public repository.

## Abbreviations

ADP, adenosine diphosphate; ALDS, Amsterdam Medical Centre linear disability score; CONSORT, Consolidated Standards of Reporting Trials; CT, computed tomography; CTA, computed tomography angiography; DICOM, Digital Imaging and Communications in Medicine; DSMB, Data Safety Monitoring Board; GCP, good clinical practice; ICH, intracerebral haemorrhage; INR, international normalised ratio; MRI, magnetic resonance imaging; mRS, modified Rankin Scale; NIHSS, National Institutes of Health Stroke Scale; SAE, serious adverse event; WMO, Dutch Medical Research involving Human Subjects Act

## Appendix

Original members of the Data Safety Monitoring Board (DSMB):

J.B. Reitsma (chairman), Department of Clinical Epidemiology, Biostatistics and Bioinformatics, Academic Medical Centre, The Netherlands

C.L. Franke, Department of Neurology; Atrium Medical Centre, Heerlen, The Netherlands

P.W. Kamphuisen, Department of Vascular Medicine; Academic Medical Centre, The Netherlands

Members of the DSMB, who performed the interim analysis:

J.G. van der Bom (chairwoman), Department of Clinical Epidemiology; Leiden University Medical Centre, Leiden, The Netherlands

A.H. Schreuder, Department of Neurology; Atrium Medical Centre, Heerlen, The Netherlands

P.W. Kamphuisen, Department of Vascular Medicine; University Medical Centre Groningen, Groningen, The Netherlands

I.W. Corten (independent statistician, who prepared the interim analysis); Clinical Research Unit of the Academic Medical Centre, The Netherlands.

Safety Committee, France

E. Touzé (chairman), Normandie Université, Université de Caen, Inserm U 919, CHU Côte de Nacre, Caen, France

D. Lasne, Laboratoire d’Hématologie, AP-HP, Hôpital Universitaire Necker-Enfants Malades, Paris, France

A. François, Etablissement Français du Sang, AP-HP, Hôpital Européen George Pompidou, Paris, France
